# Annotations

**Published:** 1910-12-17

**Authors:** 


### Annotations.

### An Advance Announcement.

One of the jobs the medical journalist has to perform pretty frequently is the pulling up of offenders against medical etiquette or against good taste in professional matters. It is not a pleasant duty (at least to an ordinarily sweet-tempered man), or a profitable or an interesting one. Disjunctively neither is it a formal protest of conservatism which means nothing, an effort of Mrs. Partington against a flowing Atlantic of new notions as to what is fitting. What it is is just the periodically necessary correction of the tendency of human nature to be careless or to obey too exclusively its instinct of self-advantage. The present instance takes the form of an advance announcement in the lay Press of a medical book, an announcement expressed very eulogistically and set out in a particularly effective "display type." No blame attaches to the publishers; their business is to sell books, not to be acquainted with the best usages of their clients' various callings. But as it is quite unlikely that a firm of the high standing of Mr. John Murray's would issue such a notice without the author's knowledge, it might be hinted to Mr. H. C. Ross that a work entitled *Induced Cell-reproduction and Cancer* can only appeal to cytologists and members of the medical and veterinary professions, and therefore that this particular puff preliminary is a mistake.

### Medical Portraiture.

The medical profession does not advertise. That is a canon of professional ethics which it would need a bold man to argue against, though we do not say that it is impossible to argue against it. But, in England at least, the profession has always been scrupulously reticent on personality. A man may be a great teacher and a leading light, and yet he may be unknown, so far as his face is concerned, to the body of his fellow-practitioners. On the Continent there is no such disinclination to have his portrait circulated among the profession. For some years past the leading medical papers in Germany have issued monthly supplements containing a photograph and a short life history of some well-known practitioner whose writings and teaching have made his name familiar to his colleagues. These artistically reproduced portraits are not only much appreciated, but have an enduring historical value which should appeal to those who are prejudiced against them on the ground that they arouse a suspicion of advertisement. With us a famous teacher, well known and honoured not only among his pupils belonging to his own school, but in the ranks of the profession, may, when he retires from the active staff of his hospital, allow his portrait to appear in the college magazine. This latter circulates only among the alumni of that particular institution with which he was immediately connected; it does not go out among the mass of the profession. And yet it is evident that a great consultant has come into contact with many practitioners during his period of activity, and that there are many men who would be glad to possess his photograph, not, as is now the case, when he is dead, but when he is still a factor to be reckoned with. The popularity of the various *Vanity Fair* cartoons—we need only instance the excellent portraits of the late Sir William Broadbent and of "Pathology," who is still, fortunately, a prominent personality in the medical world—show that such is the case. They are carefully treasured by those who are lucky enough to possess them, and in after times they will have a permanent historical value which it would be difficult to overestimate. In the circumstances it is worth considering whether in this respect, too, the German example is not worth copying in this country. We have already a series of short biographies in an extensively circulated popular manual of reference; this, at least, ought to obviate any suspicion of advertisement attaching to such a series of portraits as we suggest. A pictorial record of the profession in England is at present difficult to obtain, and we believe that if any professional paper is enterprising enough to undertake to furnish such a record it would merit, and receive, the thanks of the majority of men in the profession.

### The Beit Fellowships.

The announcement regarding the appointment of ten new Beit Scholars will not be received with much excitement by the profession at large. Hitherto the ladies and gentlemen selected to investigate the abstruse and somewhat finicky subjects that the trustees of the fund deem especially worthy of investigation have not set the scientific Thames on fire, and, as we have already pointed out, in criticising the terms of the scholarships a year ago, it is unlikely that the Beit Fund will materially advance the cause of science in its best sense, so long as these terms are not modified. It is not by increasing the already voluminous, and to a very large extent worthless, literature on medical *questiones quod libeticæ* of the day that investigators will do work of a permanent value. In the list given of the aims which the new scholars have in view, we regret to see that the clinical side has been almost entirely excluded, while the laboratory aspect has been brought into greater prominence than it deserves. Among the new scholars are Dr. Elliott, of University College, who will investigate the pathological changes in the adrenals—a huge subject which ought to yield valuable results; Dr. Atkin, of the London, who will deal with bacteriological matters; Dr. Priestley, of Sydney, who proposes to study diphtheroid organisms; Dr. Wilson, of Liverpool, who will try conclusions with the involved subject of syphilitic lipolysis; and one or two others, whose chosen subjects are equally abstruse. It is, therefore, with intense satisfaction that we note that Dr. Yates is to deal with the bacteriology of acute rheumatism—a matter that is in urgent need of revision and expert investigation. On the whole we cannot congratulate the new scholars on the subjects they have selected for research.

